# Gender- and domain-specific effects of a 6-month multidimensional exercise program on attention and verbal fluency in physically inactive older adults

**DOI:** 10.3389/fragi.2026.1769991

**Published:** 2026-06-05

**Authors:** Marlene Krumpolt, Anneke Schumacher, Lucas Sannemann, Kerstin Witte

**Affiliations:** Otto-von-Guericke-University, Magdeburg, Department of Sport, Technology and Movement Science, Magdeburg, Germany

**Keywords:** cognitive aging, cognitive functioning, executive control, gender differences, healthy aging, sport program

## Abstract

**Background:**

Regular physical activity supports cognitive functioning in older age, yet little is known about domain- and gender-specific adaptations following multidimensional training. This study examined the effects of a six-month multidimensional exercise program on attention and verbal fluency in previously inactive older adults aged 65–70 years.

**Methods:**

Participants were assigned to an intervention group (IG; n = 66, f = 44) or an inactive control group (CG; n = 31, f = 22). The six-month intervention combined structured fitness sessions and diverse recreational sports activities provided by local clubs, resulting in a multidimensional exercise program engaging physical, coordinative, cognitive, and social components. Selective and divided attention, reaction time, accuracy, and verbal fluency were assessed and analyzed using linear mixed models.

**Results:**

Significant improvements were observed for selective attention (Time × Group p = 0.034; Time × Group × Gender p = 0.031), with larger gains in men. Divided attention and phonemic fluency also improved, with women showing clearer benefits in divided attention and men in phonemic fluency. Semantic fluency remained largely stable at the group level, although within-group improvements were observed among women.

**Conclusion:**

The six-month multidimensional training program primarily reaches previously inactive older adults who would otherwise not find a suitable program and may support selective and divided attention as well as phonemic fluency. These findings highlight the relevance of gender-sensitive, multidimensional exercise interventions for maintaining cognitive functioning in later life.

## Introduction

Maintaining cognitive performance in later life is a key prerequisite for quality of life, independence, and social participation. Executive functions such as selective and divided attention, processing speed, and verbal fluency are essential for everyday competence, as they enable planning, decision-making, and the simultaneous management of multiple tasks (Diamond, 2013; Miyake and Friedman, 2012). A decline in these functions can substantially impair daily functioning and promote social isolation, as slowed speech and reduced communicative competence are often associated with negative age stereotypes, which in turn foster withdrawal and cognitive deterioration (Kotter-Grühn et al., 2009; Spuling et al., 2025).

These cognitive domains are particularly vulnerable to age-related changes that involve structural and functional adaptations in fronto-parietal networks (Raz and Rodrigue, 2006; Voelcker-Rehage and Niemann, 2013). The dorsolateral prefrontal cortex, which plays a key role in attentional control, inhibition, and action planning, shows reduced neural efficiency in older age (“last-in-first-out” principle), resulting in slower and less flexible responses (Douaud et al., 2014; Fjell and Walhovd, 2010). As selective attention declines, older adults find it increasingly difficult to distinguish relevant from irrelevant stimuli—a deficit that affects everyday life, for instance when reacting to changing environmental demands (Verhaeghen and Cerella, 2002). While automated processes are mainly governed subcortically by the basal ganglia, deliberate responses rely on increased activation of prefrontal control networks (Douaud et al., 2014). Divided attention, as part of attentional control and the ability to perform two tasks simultaneously, is also highly susceptible to age-related decline (Werner et al., 2018). Everyday situations such as walking in crowded environments or engaging in conversation while moving can be regarded as dual-task scenarios that increasingly demand cognitive resources with advancing age (Wollesen et al., 2019).

The age range between 65 and 70 years represents a particularly sensitive transitional phase in later life. This period is frequently associated with psychosocial changes such as the transition into retirement, alterations in daily structure, and shifts in social roles, which may increase vulnerability to cognitive decline and contribute to growing interindividual heterogeneity (Goelz et al., 2023). At the same time, emerging changes in attention, executive functioning, and verbal fluency are often still subclinical but functionally relevant, as even subtle impairments can affect everyday competence and autonomy (Webster-Cordero and Giménez-Llort, 2022). Focusing on this age group therefore allows the investigation of preventive interventions at a stage where cognitive plasticity remains high and training-induced adaptations are still achievable, particularly in previously inactive older adults (Galle et al., 2023). Physical activity is considered one of the most effective non-pharmacological strategies to promote cognitive health in older adults (Bherer et al., 2013; Erickson et al., 2019). Programs that integrate coordinative and cognitive demands—such as dual-task training—tend to produce stronger effects on executive functions and attention than endurance-based interventions alone (Voelcker-Rehage et al., 2011; Falck et al., 2019; Wollesen and Voelcker-Rehage, 2014; Krumpolt et al., 2025). By simultaneously activating multiple neural systems, enhancing cerebral perfusion, and promoting synaptic plasticity, such interventions facilitate both structural and functional brain adaptations (Colcombe and Kramer, 2003).

Beyond executive processes, language plays a crucial role in maintaining quality of life, social participation, and identity in older age (Huang and Davis, 2024; Huang et al., 2025). While semantic knowledge remains largely stable or may even improve through lifelong use, phonological processes—such as word retrieval and articulation—show greater age-related decline (Thornton and Light, 2006; Shafto and Tyler, 2014; Burke and Shafto, 2008). These differences stem from distinct neurocognitive mechanisms: semantics rely on stable, long-term networks, whereas phonological processes depend more on working memory and processing speed, which tend to diminish with age. Nevertheless, linguistic abilities are trainable. Cognitive-motor programs that incorporate social and communicative components have been shown to enhance both phonemic and semantic fluency (Khanna et al., 2025; Welford et al., 2023).

Sex differences also play a significant role in cognitive functioning in aging. Men often demonstrate advantages in visuospatial tasks, while women tend to excel in verbal domains (Lee et al., 2023). These differences persist into older age, although trajectories and training responses may vary between sexes (Levine et al., 2021; Venneri et al., 2024). In addition to hormonal factors, sociopsychological aspects such as socialization patterns and activity preferences contribute to these variations (Jin et al., 2023).

Increasing attention has also been directed toward concepts such as cognitive resilience and cognitive reserve. These describe the brain’s ability to maintain performance or engage compensatory mechanisms despite structural and functional changes (Stern et al., 2020; Cabeza et al., 2018). Physical activity supports these mechanisms by promoting neuroplastic adaptations and strengthening functional network connectivity (Lövdén et al., 2013). Neuroimaging and neuromodulation studies have shown that such adaptations particularly involve prefrontal and fronto-parietal regions, which remain central for attentional control and dual-task coordination (Madden et al., 2024; Park and Schott, 2025; Avelar-Pereira et al., 2017). Dual-task paradigms provide direct evidence for these effects, consistently demonstrating increased prefrontal activation in older adults, which correlates with cognitive performance and fitness (Wong et al., 2015; Baek et al., 2023; Weng et al., 2023).

Another crucial factor concerns the sustainability of such interventions. Traditional programs often show high dropout rates among older adults, especially among previously inactive individuals. In contrast, low-threshold activities embedded within community or club structures achieve higher participation rates and promote long-term behavioral changes. Participants are more likely to remain active after program completion, supporting sustained benefits for health and cognition (Schumacher et al., 2025; Krumpolt et al., 2025). Despite growing evidence, several research gaps remain. Older adults are often treated as a homogeneous group, overlooking age-specific differences. The transition from midlife to older adulthood represents a particularly sensitive period during which declines in processing speed and attentional control become more pronounced, making interventions potentially more effective (Harada et al., 2013; Perrot and Maillot, 2023). Moreover, few studies systematically assess prior physical activity, leaving it unclear to what extent previously inactive individuals benefit from training interventions. Additionally, sex-specific differences in the effectiveness of multidimensional exercise programs remain insufficiently understood (Barha et al., 2017).

Against this background, the present study addresses the following research questions: Does participation in a six-month multidimensional exercise program lead to improvements in selective attention, divided attention, and verbal fluency, and are potential effects on reaction time observable in previously inactive, healthy older adults aged 65–70 years compared with an inactive control group? Do different cognitive domains (selective attention, divided attention, verbal fluency, and reaction time) differ in their responsiveness to the intervention, and which functions show the greatest training-related improvements? Do men and women differ in their cognitive responses to the multidimensional exercise intervention, and does one sex benefit more from the training across specific cognitive domains? Overall, the findings are expected to provide important implications for personalized, sex-sensitive prevention strategies aimed at promoting cognitive health in aging.

## Materials and methods

### Participants

A total of 97 healthy older adults participated in the study and were allocated in a non-random manner to an intervention group (IG; n = 66; 21 men, 45 women) and an inactive control group (CG; n = 31; 9 men, 22 women). All participants were between 65 and 70 years of age (M = 69 years) and achieved a minimum score of 27 points on the Mini-Mental State Examination (MMSE), indicating intact global cognitive functioning (Salis et al., 2023). Educational level was categorized according to the German Qualifications Framework (DQR). Most participants were classified within higher qualification levels (levels 6–8), whereas a smaller proportion was assigned to level 5, suggesting a relatively high educational background of the sample.

Recruitment was carried out through, community networks, and public announcements. Because of logistical constraints and limited capacity within the supervised training groups, random assignment was not feasible. Therefore, allocation followed a sequential, non-randomized procedure: eligible participants were allocated to the intervention group until the maximum group size was reached; subsequently, recruited eligible participants were allocated to the control group. Allocation was based on logistical capacity rather than participant characteristics. This resulted in unequal group sizes and a gender imbalance. Given the applied and community-based nature of the study, all eligible participants were included without restriction, and this pragmatic sampling approach was considered appropriate and no *a priori* sample size calculation was performed. Control group participants received no exercise-related counseling, training offers, or structured physical activity recommendations and were instructed to maintain their usual lifestyle throughout the study period. They were contacted only for the scheduled assessment appointments. Inclusion criteria were an age between 65 and 70 years and walking independently without assistance. Physical inactivity of all participants was verified in advance by telephone interview and using the Movement and *Sport* Activity (BSA) questionnaire (Fuchs et al., 2015). The BSA questionnaire was used to verify baseline physical inactivity and was not intended to assess changes in physical activity over time. In addition to obtaining participants’ informed consent, a physician’s medical clearance was required. In contrast to many other studies, the assessment of physical activity focused on the regularity and type of participation in organized sports or structured training groups within clubs. Everyday activities such as housework, gardening, walking, or cycling for transportation or shopping were not considered exclusion criteria. Participants were classified as physically inactive if they had not engaged in regular structured exercise for more than 2 years. Exclusion criteria included neurological or psychiatric disorders, severe internal or orthopedic limitations, and a diagnosis of dementia. All participants provided written informed consent prior to participation.

The study was conducted in accordance with the Declaration of Helsinki and approved by the Ethics Committee of the Otto von Guericke University Magdeburg (No. 3/22). The trial was registered in the German Clinical Trials Register (DRKS00030853; registered 14 December 2022).

### Intervention

The intervention consisted of a six-month multidimensional exercise program that included one 90-min fitness session and one 90-min recreational sports session per week, both supervised by certified instructors from the project team and local sports clubs in Magdeburg. The fitness sessions focused on coordination, strength, flexibility, and endurance, while the recreational sessions included a variety of disciplines such as dancing, racket sports, and ball games. Both playful and technical exercises were used to promote engagement and skill development. The training plan was set for each participant and was identical for everyone, who trained together as a cohesive cohort. The sport-specific modules followed a predefined weekly rotation, so that everyone went through the same sequence of activities. No prior knowledge or specific fitness level was required, as the program aimed to introduce participants to various sports through basic techniques and structured, playful exercises. The exercises were individually adapted through graded tasks, variations in equipment, and optional adjustments to intensity to ensure feasibility and safety. Participation was documented via attendance lists. Participants with an attendance rate of less than 75% were excluded from the final analysis.

The program was specifically tailored to previously physically inactive older adults. Training intensity was progressively increased over the 24-week intervention period, particularly through adjustments to task complexity, coordination requirements, and exercise duration. Although standardized training manuals ensured a high degree of comparability between groups, the focus of the multimodal program was on variability rather than strictly defined intensity zones. In addition, theoretical and practical modules on healthy nutrition were integrated into the program (Schumacher et al., 2025). The interdisciplinary approach aimed to promote both physical and cognitive activity while simultaneously strengthening social interaction. Cognitive and motor assessments were conducted before (T0) and immediately after the intervention (T1).

### Reaction time

Reaction time was assessed using the Choice Reaction Time test (RTS4) from the Vienna Test System (Schuhfried, 2023). This instrument is considered a reliable and validated indicator of processing speed in older adults. Choice reaction time is a key marker of the aging process, as such tasks are regarded as particularly sensitive in cognitive aging research. Beyond pure motor execution, they also capture stimulus–response mapping and decision-making processes (Chintapalli and Romero-Ortuno, 2020).

### Dual-task assessment (divided attention)

Participants completed a dual-task protocol using the OptoGait system (software version 1.12, Microgate). Three conditions were assessed:

- Single Task–Motor ( $v_{ST\_M}$ ): straight-ahead walking over 100 m at a self-selected pace (Gordt et al., 2019; Röll, 2021).
- Single Task–Cognition ( $n_{ST\_C}$ ): serial subtraction (Goh et al., 2021).
- Dual Task (DT): simultaneous performance of both tasks.

For each condition, walking performance and cognitive performance were recorded. Walking performance was indexed by walking speed (v), and cognitive performance by the number of correct calculation steps (c). Dual-task costs were computed separately for motor ( $DTC_{M}\left(v\right)$ and cognitive ( $DTC_{C\;}\left(n\right)$ performance according to Equations 1, 2. To express dual-task ability independent of individual task prioritization, the mean DTC was calculated according to Equation 3 (Beurskens and Bock, 2013). Lower mean DTC values indicate better divided attention (Brustio et al., 2017).

$$DTC_{M}\left(v\right)\left[\%\right]=\frac{v_{ST\_M}-v_{DT}}{v_{ST\_M}}\times 100$$ (1)

$$DTC_{C\;}\left(n\right)\left[\%\right]=\frac{n_{ST\_C}-n_{DT}}{n_{ST\_C}}\times 100$$ (2)

$$mean\;DTC\;\left[\%\right]=\frac{\left[DTC_{M}\left(v\right)\right]+\left[DTC_{C\;}\left(n\right)\right]}{2}$$ (3)

Key: = v walking speed; = n number of correct calculation steps.

### Stroop test (selective attention)

Selective attention and inhibitory control were assessed using a standardized version of the Stroop Test (S7) from the Vienna Test System (Stroop, 1935; Schuhfried, 2023). This test is an established and widely used instrument for measuring executive functions and selective attention in older adults (Scarpina and Tagini, 2017).

### Verbal fluency

Verbal fluency was assessed using the Regensburger Word Fluency Test (RWT) as a measure of divergent thinking (Aschenbrenner et al., 2001). The RWT includes both lexical and semantic subtests (formal–lexical alternation and semantic–categorical alternation). It is considered a valid instrument for assessing executive language processes and semantic retrieval ability, even in older adults (Amunts et al., 2021).

### Accuracy

Accuracy was operationalized differently across tasks in accordance with their scoring procedures. For the Stroop task, accuracy was defined as the number of false responses (N), with lower values indicating better performance. In contrast, for the RTS4 task, accuracy was quantified as the number of correct responses out of 16 trials, with higher values reflecting better performance.

All assessments were conducted individually in a standardized laboratory setting. Pre- and post-intervention measurements (T0 and T1) were performed within a defined time window. Although testing did not take place at exactly the same time of day for all participants, efforts were made to assess individuals at similar times (e.g., morning sessions) across both measurement points to reduce circadian variability. A fixed testing protocol was applied for all participants in the same order: (1) reaction time (computer-based), (2) verbal fluency (paper–pencil), (3) Stroop test (computer-based), and (4) dual-task assessment using the OptoGait system. All assessments were conducted using standardized instructions by trained project staff to ensure consistency across participants and measurement time points.

All of these tests are feasible for untrained older adults (Table 1).

**TABLE 1:** Applied test procedures and associated cognitive domains.

Test procedure	Task description	Capability area
STROOP (wiener testsystem)	Measurement of selective attention and inhibition. Naming interference (automated): RT difference between reading interference and baseline, reflecting performance of the automated process. Reading interference (conscious): RT difference between naming interference and baseline, reflecting controlled, conscious processing. Accuracy: Number of false responses (N)	Selective attention/inhibitory control
Reaction test (wiener testsystem)	Choice reaction task assessing motor reaction time (ms) and cognitive processing time (ms). Accuracy: Correct responses out of 16 trials	Processing speed/psychomotor speed
Dual-task (OptoGait)	Single task – walking: gait speed (m/s). Single task – cognitive: serial subtraction. Dual task: both simultaneously. Dual-task costs (DTC, %) for gait and cognition and mean DTC were calculated	Divided attention/cognitive-motor interference
Verbal fluency test (RWT)	Phonemic: Number of words with given initial letter (2 min). Semantic: number of words within a category (2 min). The number of correct words (N) was recorded for each condition	Executive function/verbal fluency

### Statistical analyses

All statistical analyses were performed using R (version 4.5.1) and SPSS (version 29.0). The level of significance was set at α = 0.05. Due to unequal group sizes, partially small subgroups, and violations of homogeneity of variance, linear mixed-effects models (LMMs) with random intercepts at the participant level were applied. Fixed effects included time (pre/post), group (intervention vs. control), sex, and all interaction terms (time × group × sex).

Linear mixed-effects models were fitted in R using the lme4 and lmerTest packages, with Type III tests of fixed effects obtained via the car package. For outcome variables with non-normally distributed residuals (e.g., Mean DTC, Stroop interference measures), a Box–Cox transformation was applied prior to model estimation to meet the assumptions of parametric testing (Blum et al., 2022).

To further explore significant effects, post-hoc analyses were conducted. Within-group changes from pre-to post-test were examined using paired-samples t-tests, while between-group comparisons of change scores (Δ = post − pre) were analyzed using Welch’s t-tests, which are robust to unequal variances and unequal sample sizes. To control for inflation of Type I error due to multiple testing, the Holm correction was applied (Holland and Copenhaver, 1987).

Effect sizes for LMMs were quantified using marginal and conditional R^2^ (Johnson, 2014). For pairwise comparisons, Cohen’s d and Hedges’ g were calculated. Estimated marginal means and model-based contrasts were obtained using the emmeans package (Lenth et al., 2025). Additionally, nonparametric tests (e.g., Wilcoxon signed-rank tests) were conducted as robustness checks where appropriate. All analyses followed a hypothesis-driven approach.

## Results

### Selective attention (conscious/automated; accuracy)

Descriptive statistics indicated reduced reaction times from T0 to T1 in both Stroop components within the IG (lower values = better performance), whereas the CG remained largely stable (Table 2). LMM analyses revealed a significant main effect of Time for the conscious component (χ^2^(1) = 4.376, p = 0.036, R^2^_mar_g = 0.05), as well as significant Time × Group (χ^2^(1) = 4.493, p = 0.034, R^2^_mar_g = 0.05) and Time × Group × Gender interaction effects (χ^2^(1) = 4.650, p = 0.031, R^2^_mar_g = 0.05). For the automated component, a significant main effect of Time was also observed (χ^2^(1) = 11.17, p = 0.001, R^2^_mar_g = 0.027). Regarding Stroop Accuracy, a significant Time effect was found for the conscious component (χ^2^(1) = 8.297, p = 0.004, R^2^_mar_g = 0.064) (Table 3). Between-group comparisons of change scores (Δ = T0–T1) revealed no significant difference between IG and CG for the conscious component (t(40.34) = −0.461, p = 0.647; g = −0.119). However, IG men performed significantly better than CG men (t(19.43) = −2.623, p = 0.034; g = −0.93), whereas no significant difference was found among women (t(31.26) = 0.518, p = 0.485; g = 0.168). For the automated component and Stroop Accuracy, no significant between-group effects emerged (all p ≥ 0.45; g values small in magnitude) (Table 4). Within-group analyses confirmed significantly shorter reaction times in the IG for both the conscious (t(65) = 2.641, p = 0.010; d = −0.325) and automated Stroop components (t(65) = 2.289, p = 0.025; d = −0.282), along with higher Stroop Accuracy for the conscious (t(65) = 3.959, p = 0.001; d = −0.450) and trend-level improvement for automated components (t(65) = 1.78, p = 0.08; d = −0.22) (Table 5). Subgroup analyses showed that IG men exhibited significantly faster reaction times (conscious: t(20) = 2.236, p = 0.037; d = −0.488; automated: t(20) = 3.091, p = 0.006; d = −0.675), whereas IG women primarily improved in accuracy (t(44) = 2.694, p = 0.010; d = −0.402). No significant changes were observed in the CG (all p ≥ 0.18) (Table 6).

**TABLE 2:** Results of executive function from baseline (T0) and post-test (T1) for the intervention group (IG) and control group (CG).

Cognitive Performance Parameters	IG n = 66 (m = 21; f = 45)						CG n = 31 (m = 9; f = 22)					
	T0 M±SD			T1 M±SD			T0 M±SD			T1 M±SD		
	m	f	Total	m	f	Total	m	f	Total	m	f	Total
Selective attention												
Conscious (sec) ↓	1.12 ± 0.23	1.03 ± 0.15	1.06 ± 0.18	1.04 ± 0.18	1.00 ± 0.15	1.02 ± 0.16	1.01 ± 0.13	0.99 ± 0.19	1.00 ± 0.18	1.07 ± 0.17	0.94 ± 0.29	0.98 ± 0.27
Accuracy (N) ↓	7.4 ± 8.3	2.4 ± 2.3	3.9 ± 4.8	1.3 ± 1.8	1.3 ± 1.9	1.3 ± 1.8	2.3 ± 2.1	3.1 ± 3.6	2.8 ± 3.2	1.9 ± 1.9	2.1 ± 2.7	2.0 ± 2.5
Automated (sec) ↓	0.99 ± 0.22	0.95 ± 0.17	0.96 ± 0.19	0.92 ± 0.16	0.95 ± 0.17	0.94 ± 0.17	1.00 ± 0.33	0.89 ± 0.19	0.93 ± 0.24	0.97 ± 0.17	0.89 ± 0.21	0.91 ± 0.19
Accuracy (N) ↓	1.6 ± 2.6	2.1 ± 3.3	1.9 ± 3.1	1.1 ± 1.5	1.4 ± 1.9	1.3 ± 1.7	3.2 ± 8.2	3.2 ± 4.8	3.2 ± 5.8	1.2 ± 0.97	1.9 ± 3.4	1.7 ± 2.9
Divided attention mean DTC (%) ↓	20.4 ± 16.0	24.5 ± 15.0	23.2 ± 15.4	10.5 ± 13.3	15.7 ± 13.6	14.0 ± 13.6	15.5 ± 7.8	20.7 ± 17.9	19.1 ± 15.7	14.7 ± 12.9	20.9 ± 21.4	19.1 ± 19.3
Verbal fluency (N)												
Phonemic ↓	16.5 ± 5.2	18.8 ± 5.2	18.0 ± 5.3	19.4 ± 4.7	20.4 ± 6.5	20.1 ± 5.9	18.0 ± 5.2	20.9 ± 4.6	20.0 ± 5.9	17.7 ± 4.1	19.8 ± 5.9	19.2 ± 5.4
Semantic ↓	20.1 ± 5.3	21.2 ± 3.7	20.9 ± 4.3	19.3 ± 4.1	23.0 ± 5.5	21.8 ± 5.4	21.0 ± 3.8	22.9 ± 3.7	22.3 ± 3.8	18.0 ± 4.5	22.7 ± 3.5	21.3 ± 4.3
Reaction time												
Motor (ms) ↓	336.6 ± 68.4	348.3 ± 95.2	343.3 ± 89.4	328.19 ± 50.5	338.1 ± 79.9	334.7 ± 72.9	361.4 ± 120.5	360.1 ± 119.0	359.7 ± 121.2	350.3 ± 64.5	354.2 ± 107.3	351.9 ± 94.2
Processing (ms) ↓	330.4 ± 73.6	345.7 ± 99.3	340.6 ± 91.7	323.2 ± 50.7	334.1 ± 80.9	330.5 ± 72.8	355.7 ± 133.2	358.2 ± 121.7	358.5 ± 123.9	339.2 ± 49.3	350.6 ± 107.4	345.3 ± 93.4
Accuracy (N) ↓	15.9 ± 0.4	15.87 ± 0.4	15.88 ± 0.4	16.0 ± 0.0	15.96 ± 0.2	15.97 ± 0.2	15.89 ± 0.3	16.0 ± 0.0	15.97 ± 0.3	16.0 ± 0.0	15.91 ± 0.3	15.94 ± 0.3

↑ and ↓ indicate whether a high or low test score reflects optimal performance, M mean, SD standard deviation, n sample size, m male, f female, N number of responses.

**TABLE 3:** Results of the comparison of the interaction intervention group (IG) and control group (CG).

Cognitive Performance Parameters	Group			Time			Group × Time			Group × Time × Sex		
	Effect size			Effect size			Effect size			Effect size		
Selective attention	Chi^2^ (df)	p	η^2^	Chi^2^ (df)	p	η^2^	Chi^2^ (df)	p	η^2^	Chi^2^ (df)	p	η^2^
Conscious (sec)	1.87 (1)	0.171	0.010	4.37(1)	**0.036**	0.023	4.49 (1)	**0.034**	0.024	4.65 (1)	**0.031**	0.024
			R^2^			R^2^			R^2^			R^2^
			0.05			0.05			0.05			0.05
Accuracy (N)	0.633 (1)	0.426	0.03	8.29(1)	**0.004**	0.043	1.440 (1)	0.230	0.08	0.915 (1)	0.339	0.05
			R^2^			R^2^			R^2^			R^2^
			0.064			0.064			0.064			0.064
Automated (sec)	0.025 (1)	0.875	0.000	11.1(1)	**0.001**	0.057	0.440 (1)	0.507	0.002	0.206 (1)	0.650	0.001
			R^2^			R^2^			R^2^			R^2^
			0.027			0.027			0.027			0.027
Accuracy (N)	1.60 (1)	0.21	0.009	0.30 (1)	0.59	0.002	0.91 (1)	0.34	0.005	0.25	0.61	0.001
			R^2^			R^2^			R^2^			R^2^
			0.001			0.001			0.001			0.001
Divided attention mean DTC (%)	0.640 (1)	0.424	0.003	7.12 (1)	**0.008**	0.037	1.80 (1)	0.179	0.010	0.000 (1)	0.998	0.00
			R^2^			R^2^			R^2^			R^2^
			0.079			0.079			0.079			0.079
Verbal fluency (N)	Chi^2^ (df)	p	η^2^	Chi^2^ (df)	p	η^2^	Chi^2^ (df)	p	η^2^	Chi^2^ (df)	p	η^2^
Phonemic	0.482 (1)	0.488	0.003	7.18 (1)	**0.007**	0.037	2.86 (1)	0.090	0.015	0.107 (1)	0.744	0.001
			**R** ^**2**^			**R** ^**2**^			**R** ^**2**^			**R** ^**2**^
			0.045			0.045			0.045			0.045
Semantic	0.303 (1)	0.582	0.002	0.689 (1)	0.582	0.004	1.784 (1)	0.182	0.010	0.014 (1)	0.907	0.000
			R^2^			R^2^			**R** ^**2**^			R^2^
			0.011			0.011			0.011			0.011
Reaction time	Chi^2^ (df)	p	η^2^	Chi^2^ (df)	p	η^2^	Chi^2^ (df)	p	η^2^	Chi^2^ (df)	p	η^2^
Motor (ms)	0.07 (1)	0.787	0.00	1.14 (1)	0.286	0.001	0.06 (1)	0.808	0.001	0.35 (1)	0.557	0.002
			R^2^			R^2^			R^2^			R^2^
			0.006			0.006			0.006			0.006
Processing (ms)	0.49 (1)	0.482	0.003	0.58 (1)	0.445	0.003	0.60 (1)	0.438	0.004	0.04 (1)	0.847	<0.001
			R^2^			R^2^			R^2^			R^2^
			0.004			0.004			0.004			0.004
Accuracy (N)	0.02 (1)	0.888	0.002	1.18 (1)	0.277	0.004	0.01 (1)	0.921	0.0001	1.05 (1)	0.306	0.002
			R^2^			R^2^			R^2^			R^2^
			0.031			0.031			031			031

Linear mixed models LMM, R^2^ values represent marginal coefficients of determination. η^2^ values are provided as approximate effect size indicators and should be interpreted with caution, as η^2^ is not a standard effect size measure for linear mixed models, statistically significant: p ≤ 0.05. Model significance was tested using likelihood-ratio tests (χ^2^) based on −2 log-likelihood comparisons estimated with maximum likelihood (ML). Bold values indicate statistically significant results (p ≤ 0.05).

**TABLE 4:** Results of differences over time between the intervention group (IG) and control group (CG).

Cognitive performance	Between-group effects (IG vs. CG)▲								
	Total			Male			Female		
Selective attention	welch(df)	p*	g	welch(df)	p*	welch(df)	welch(df)	p*	welch(df)
Conscious (sec)	−0.461 (40.34)	0.647	−0.119	−2.623 (19.43)	**0.034**	−0.93	0.518 (31.26)	0.485	0.168
Accuracy (N)	−0.698 (41.45)	0.488	−0.159	−1.425 (11.23)	0.166	−0.445	−0.077 (29.43)	0.94	−0.022
Automated (sec)	−0.538 (63.66)	0.593	−0.122	−0.429 (13.23)	0.675	−0.17	−0.162 (43.94)	0.872	−0.04
Accuracy (N)	0.77 (44.17)	0.45	0.21	0.54 (10.63)	0.60	0.29	0.51 (31.06)	0.61	0.15
Divided attention mean DTC (%)	−2.40 (77.24)	**0.02**	−0.528	−1.554 (17.83)	0.179	−0.592	−1.866 (53.66)	**0**.**039**	−0.493
Verbal fluency (N)	welch(df)	p*	g	welch(df)	p*	g	welch(df)	p*	g
Phonemic	2.382 (78,46)	**0.021**	0.557	2.178 (15.91)	**0.043**	0.767	2.055 (55.62)	**0**.**044**	0.609
Semantic	2.239 (78.73)	**0.028**	0.449	1.691 (16.71)	0.109	0.625	1.831 (57.58)	0.067	0.431
Reaction time	welch(df)	p*	g	welch(df)	p*	g	welch(df)	p*	g
Motor (ms)	−0.25 (78.82)	0.803	−0.05	−1.27 (17.84)	0.224	−0.49	−0.55 (58.43)	0.588	−0.13
Processing (ms)	−0.02 (79.36)	0.98	−0.01	0.66 (12.84)	0.51	0.22	−0.49 (55.98)	0.62	−0.12
Accuracy (N)	1.55 (69.42)	0.125	0.29	−0.11 (25.78)	0.921	−0.04	1.92 (45.65)	0.085	0.42

p*Holm corrector, statistically significant: p ≤ 0.05, welch test, g effect size. Bold values indicate statistically significant results (p ≤ 0.05).

**TABLE 5:** Results of time analysis of intervention group (IG) and control group (CG).

Cognitive performance parameters	Within-group effects					
	IG			CG		
Selective attention	t(df)	p*	d	t(df)	p*	d
Conscious (sec)	2.641 (65)	**0.010**	−0.325	0.576 (30)	0.569	−0.103
Accuracy (N)	3.959 (65)	**0.001**	−0.450	1.384 (30)	0.184	−0.244
Automated (sec)	2.289 (65)	**0.025**	−0.282	0.740 (30)	0.465	−0.133
Accuracy (N)	1.78 (65)	0.08	−0.22	1.42 (30)	0.166	−0.26
Divided attention mean DTC (%)	4.403 (65)	**<0.001**	−0.542	0.006 (30)	0.995	−0.001
Verbal fluency (N)	t(df)	p*	d	t(df)	p*	d
Phonemic	−3.504 (65)	**0.001**	0.431	0.801 (30)	0.430	−0.144
Semantic	−1.684 (65)	0.097	0.207	1.590 (30)	0.142	−0.271
Reaction time	t(df)	p*	d	t(df)	p*	d
Motor (ms)	1.94 (65)	0.056	−0.24	0.97 (30)	0.338	−0.17
Processing (ms)	1.88 (65)	0.064	−0.23	1.07 (30)	0.29	−0.19
Accuracy (N)	−1.62 (65)	0.109	0.20	0.57 (30)	0.572	−0.10

p*Holm Corrector, t-test, statistically significant: p ≤ 0.05, d effect size. Bold values indicate statistically significant results (p ≤ 0.05).

**TABLE 6:** Results of time analysis of male and female intervention group (IG); male and female control group (CG).

Cognitive performance parameters	Within-group effects											
	Male IG			Male CG			Female IG			Female CG		
Selective attention	t(df)	p*	d	t(df)	p*	d	t(df)	p*	d	t(df)	p*	d
Conscious (sec)	2.236 (20)	**0.037**	−0.488	−1.554 (8)	0.159	0.518	1.582 (44)	0.121	−0.236	1.101 (21)	0.283	−0.235
Accuracy (N)	2.47 (20)	**0.022**	−0.540	0.686 (8)	0.512	−0.229	2.694 (44)	**0.01**	−0.402	1.195 (21)	0.246	−0.255
Automated (sec)	3.091 (20)	**0.006**	−0.675	0.792 (8)	0.451	−0.264	0.543 (44)	0.59	−0.081	0.145 (21)	0.886	−0.031
Accuracy (N)	0.78 (20)	0.44	−0.17	0.72 (8)	0.49	−0.24	1.59 (44)	0.12	−0.24	1.28 (21)	0.22	−0.27
Divided attention mean DTC (%)	3.001 (20)	**0.007**	−0.655	0.165 (8)	0.873	−0.055	3.318 (44)	**0**.**002**	−0.495	−0.073 (21)	0.943	0.016
Verbal fluency (N)	t(df)	p*	d	t(df)	p*	d	t(df)	p*	d	t(df)	p*	d
Phonemic	−3.11 (20)	**0.005**	0.679	0.199 (8)	0.847	−0.066	−2.212 (44)	**0**.**032**	0.330	0.838 (21)	0.412	−0.179
Semantic	0.972 (20)	0.342	−0.212	2.811 (8)	**0**.**023**	−0.937	−2.416 (44)	**0.020**	0.360	0.28 (21)	0.782	−0.06
Reaction time	t(df)	p*	d	t(df)	p*	d	t(df)	p*	d	t(df)	p*	d
Motor (ms)	1.12 (20)	0.276	−0.24	0.77 (8)	0.463	−0.26	1.74 (44)	0.078	−0.26	0.44 (21)	0.663	−0.09
Processing (ms)	0.82 (20)	0.42	−0.18	0.67 (8)	0.52	−0.22	1.61 (44)	0.12	−0.24	0.71 (21)	0.49	−0.15
Accuracy (N)	−1.0 (20)	0.329	0.22	−1.0 (8)	0.347	0.33	0.1,27 (44)	0.209	0.19	1.45 (21)	0.162	−0.31

p*Holm Corrector, t-test, statistically significant: p ≤ 0.05, d effect size. Bold values indicate statistically significant results (p ≤ 0.05).

### Divided attention (mean DTC %)

Mean DTC decreased from T0 to T1 in the IG, while remaining unchanged in the CG (Table 2). The LMM revealed a significant main effect of Time (χ^2^(1) = 7.127, p = 0.008, R^2^_mar_g = 0.079) (Table 3). Between-group comparisons indicated a significant difference favoring the IG (t(77.24) = −2.40, p = 0.020; g = −0.528). Gender-specific analyses showed that this difference was not significant among men (t(17.83) = −1.554, p = 0.179; g = −0.592), but was significant among women (t(53.66) = −1.866, p = 0.039; g = 0.493) (Table 4).

Within-group analyses revealed a significant reduction in Mean DTC in the IG (t(65) = 4.403, p < 0.001; d = −0.542), which was evident in both men (t(20) = 3.001, p = 0.007; d = −0.655) and women (t(44) = 3.318, p = 0.002; d = −0.495). No significant changes occurred in the CG (all p ≥ 0.873) (Tables 5, 6).

### Verbal fluency (phonemic/semantic)

Phonemic fluency increased from T0 to T1 in the intervention group, whereas a slight decline was observed in the control group. For semantic fluency, only minor improvements were observed in the intervention group, while the control group again showed slight decreases (Table 2). The LMM confirmed a significant main effect of Time for phonemic fluency (χ^2^(1) = 7.188, p = 0.007, R^2^_mar_g = 0.045), but not for semantic fluency (χ^2^(1) = 0.689, p = 0.582, R^2^_mar_g = 0.011) (Table 3). Between-group comparisons revealed significantly greater phonemic gains in the IG compared with the CG (total sample: t(78.46) = 2.382, p = 0.021; g = 0.557; men: t(15.91) = 2.178, p = 0.043; g = 0.767; women: t(55.62) = 2.055, p = 0.044; g = 0.609). For semantic fluency, a significant overall effect was found (t(78.73) = 2.239, p = 0.028; g = 0.449), but subgroup analyses were non-significant (men: p = 0.109; women: p = 0.067) (Table 4). Within-group analyses supported these findings: phonemic fluency significantly increased in the IG (t(65) = −3.504, p = 0.001; d = 0.431), whereas semantic fluency did not (t(65) = −1.684, p = 0.097) (Table 5). Gender-specific within-group analyses revealed significant improvements in phonemic fluency over time in both men and women of the IG. Specifically, men in the IG showed a significant increase in phonemic fluency (t(20) = −3.112, p = 0.005; d = 0.679), while women in the IG also demonstrated significant improvements (t(44) = −2.212, p = 0.032; d = 0.330). In addition, women in the IG exhibited a significant within-group increase in semantic fluency (t(44) = −2.416, p = 0.020; d = 0.360) (Table 6).

No significant changes were detected in the CG (all p ≥ 0.142) (Tables 5, 6).

### Reaction time (motor/processing) and RT accuracy

Descriptive data indicated only minor changes across all groups. Mean motor and cognitive reaction times, as well as RT accuracy, remained largely stable (Table 2). LMM analyses revealed non-significant results (p = >0.286) (Table 3). Between-group comparisons revealed no significant differences between IG and CG (motor RT: t(78.82) = −0.25, p = 0.803, g = −0.05; processing RT: t(79.36) = −0.02, p = 0.98, g = −0.01; RT accuracy: t(69.42) = 1.55, p = 0.125, g = 0.29) (Table 4). Within the IG, only non-significant trends were observed (motor RT: t(65) = 1.94, p = 0.056 and no significant changes were found in the CG (Tables 5, 6).

### Summary

**FIGURE 1:**
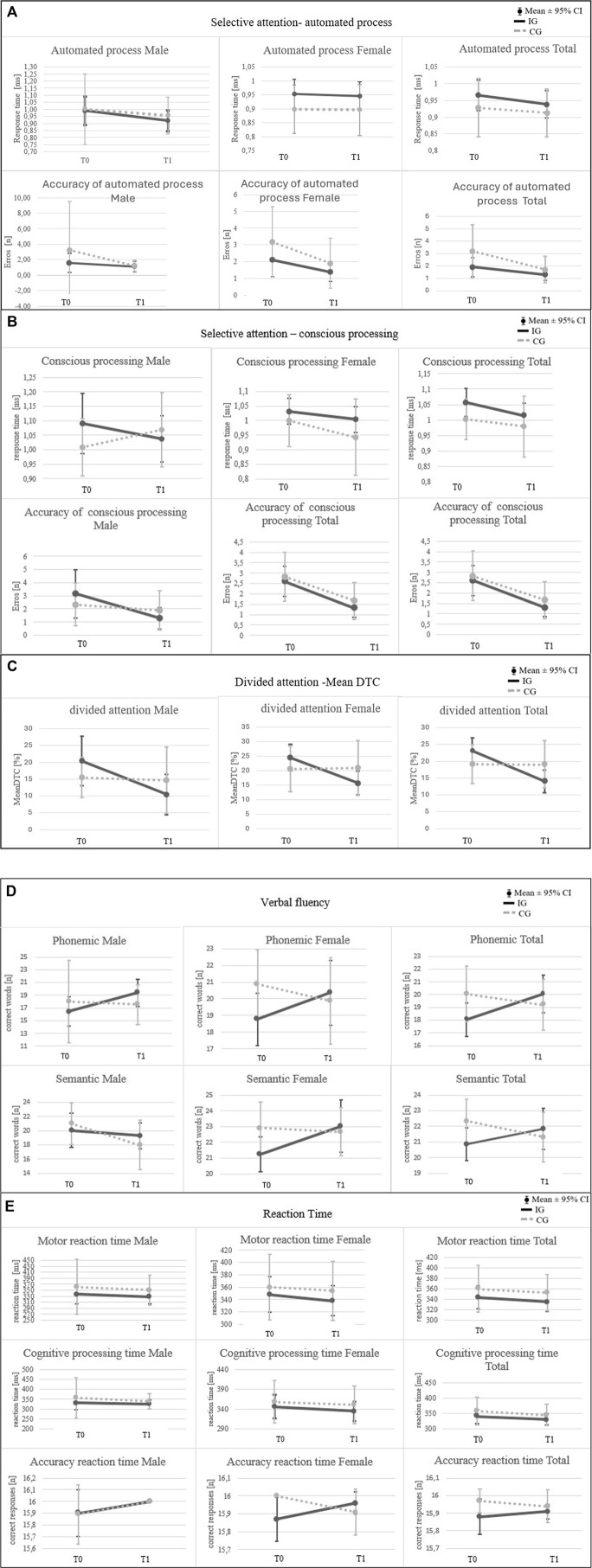
**(A)** Time*group effects for the automated process in selective attention of the IG (male and female) and CG (male and female), total (including gender). Note: Lower value indicates better performance. **(B)** Time*group effects for the conscious process in selective attention of the IG (male and female) and CG (male and female), total (including gender). Note: Lower value indicates better performance. **(C)** Time*group effects for the mean Dual-Task-Cost (mean DTC) in divided attention of the IG (male and female) and CG (male and female), total (including gender). Note: Lower value indicates better performance. **(E)** Time*group effects for the reaction time of the IG (male and female) and CG (male and female), total (including gender). Note: Lower value indicates better performance by reaction time; Higher value indicates better performance by accuracy. **(D)** Time*group effects for the verbal fluency (phonemic and semantic) of the IG (male and female) and CG (male and female), total (including gender). Note: Higher value indicates better performance by verbal fluency.

Overall, the multidimensional training program primarily improved selective attention, divided attention, and phonemic verbal fluency. Both descriptive and inferential analyses supported these effects and showed partial gender specificity, with larger performance gains in men for selective attention and phonemic fluency. Semantic verbal fluency remained largely stable, although women in the intervention group showed significant within-group improvements. Motor and cognitive reaction times, as well as RT accuracy, did not change significantly. No systematic changes were observed in the control group throughout the study period. Analyses of cognitive performance yielded differentiated effects across time, group, and interaction terms, as well as gender-specific differences. Early in the study, the groups showed minor differences in the variables that should be taken into account when interpreting interactions (Figures 1A–E). To account for these differences at the start of the study, mixed-effects linear models incorporating time × group interactions were used.

Interactions between IG and CG are illustrated in Figures 1A–E.

## Discussion

The present study examined the effects of a six-month multidimensional exercise program on attentional and language-related cognitive functions in previously inactive, healthy older adults aged 65–70 years. By combining coordinative and cognitive elements with broad-based recreational sports activities within a low-threshold, community-based training approach, the intervention was designed to target executive control processes that are particularly relevant for everyday functioning in later life. Using a controlled pre–post design and linear mixed-model analyses, changes in selective attention, divided attention, verbal fluency, and reaction time were assessed, with a specific focus on domain-specific responsiveness and gender-related change patterns.

Against this methodological background, the present multidimensional exercise program demonstrated domain-specific cognitive effects in previously inactive older adults aged 65–70 years. Improvements were observed primarily in selective attention, divided attention (dual-task costs), and phonemic verbal fluency, whereas semantic fluency and simple reaction time remained largely stable. Gender-specific analyses further indicated distinct response patterns, with men showing greater gains in executive control processes and phonemic fluency, while women exhibited stronger improvements in accuracy and a more pronounced reduction in dual-task costs compared with the control group.

Given the six-month intervention period and the initially inactive profile of participants, these findings may reflect early-stage neurocognitive adaptations induced by the multidimensional training approach.

### Attention and executive control

The improvement in attention aligns with previous findings indicating that multidimensional training programs combining coordinative, cognitive, and social elements can support executive functioning in older adults (Voelcker-Rehage and Niemann, 2013; Falck et al., 2019). Processing speed and attentional control are especially prone to age-related decline but also respond well to targeted interventions (Harada et al., 2013; Colcombe and Kramer, 2003). Dual-task paradigms are sensitive to cognitive–motor interference, which increases with age due to structural and functional changes in fronto-parietal networks (Ross et al., 2021). Neuroimaging studies show that dual-task conditions in older adults often involve enhanced prefrontal activation, interpreted as compensatory recruitment (Avelar-Pereira et al., 2017). The reduction in mean DTC in the intervention group may therefore indicate more efficient integration of cognitive and motor processes and improved resource allocation.

The three-way interaction between group, time, and gender observed in the linear mixed model further supports the domain-specific nature of these adaptations, prompting separate discussion of attention, accuracy, and verbal fluency outcomes.

Although both genders showed within-group improvements, significant between-group differences (IG vs. CG) emerged mainly among women, who enhanced dual-task performance compared to controls. Men improved as well, but these changes did not reach between-group significance. This pattern may suggets that women showed clearer intervention-specific benefits in divided attention relative to the control group. However, this interpretation should be made cautiously, as the very small male control group (n = 9) may have limited statistical power to detect between-group effects in men. Previous work reported gender differences in dual-task paradigms, with women often showing higher baseline mean DTC and thus greater training potential (Peterson, 2022; Elshorbagy et al., 2024). Other studies found stronger mean DTC increases in men, possibly due to differing prioritization or cognitive–motor coupling strategies (Christova et al., 2025). Wollesen and Voelcker-Rehage (2014) highlighted that multidimensional programs may enhance cognitive–motor coupling and reveal gender-related differences in adaptive flexibility. Our results position themselves between these findings: women improved more relative to controls, while men’s progress remained within-group.

Mechanistically, these effects could reflect enhanced activation and efficiency within fronto-parietal networks (Campbell et al., 2012). Meta-analyses confirm that dual-task-specific training can reduce mean DTC and improve mobility and executive functioning (Ali et al., 2022; Ercan Yildiz et al., 2024). Thus, the mean DTC reduction might indicate better resource allocation and control-network efficiency (Erickson et al., 2019). Although the explained variance was small to moderate (R^2^marg ≈0.05), the direction and consistency of effects suggest meaningful domain-specific improvements in attention and executive control. These findings extend previous research by suggesting that previously inactive older women could particularly benefit from multidimensional exercise.

### Conscious vs. automated attention processes

Notably, the intervention improved consciously controlled attentional processes, which rely on dorsolateral prefrontal and anterior cingulate networks. These processes are typically less plastic and harder to modify in later life due to reduced network flexibility and fronto-parietal efficiency (Forte et al., 2024; Cipriani et al., 2025). The observed improvements underscore the potential of complex, multidimensional training to enhance executive attentional control in older adulthood. Importantly, these effects were primarily driven by within-group improvements in the intervention group, whereas between-group differences remained limited.

Recent neuroimaging studies indicate that physically active older adults exhibit lower yet more efficient prefrontal activation during executive tasks—suggesting training-induced optimization (Song et al., 2025). The present performance gains could therefore reflect increased efficiency in consciously controlled attention. Cipriani et al. (2025) further showed that age-related executive changes are accompanied by functional and physiological adaptations, supporting the relevance of training-induced neuroplasticity.

Interestingly, men improved not only in automated but also in consciously controlled attention. Such processes are closely linked to everyday functioning, including safe navigation of traffic or coordinating multiple task sequences (Holtzer et al., 2014). These results may suggest that men—often less engaged in preventive interventions—could benefit from programs demanding higher executive control (Barha et al., 2017; Lee et al., 2023).

### Accuracy and strategic adjustments

Beyond reaction time, the intervention group showed significant accuracy gains in the Stroop task. Women, in particular, responded more accurately in the conscious condition, even without parallel reductions in reaction time. This pattern might indicate a speed–accuracy trade-off typical of older adults (Verhaeghen and Cerella, 2002). The results also support the idea that women tend to prioritize precision under higher cognitive demands (Bianco et al., 2020).

Between-group differences were non-significant, suggesting that accuracy improvements occurred mainly within the intervention group. Such gains might reflect strategic adaptations, such as more conservative response tendencies or improved response selection. Together, these results highlight dimension-specific training effects—women may optimize accuracy, whereas men focus on speed (Lee et al., 2023).

### Phonemic and semantic verbal fluency

The program particularly enhanced phonemic fluency, consistent with studies showing that phonemic processes—time-sensitive retrieval of word forms and phonological structures—are more affected by aging than semantic processes, which rely on more stable associative networks (Shafto et al., 2014; Ouyang et al., 2020; Frankenberg et al., 2023). While semantic fluency tends to remain stable in healthy aging, phonemic fluency depends on processing speed, working memory, and executive control, making it more age-sensitive (Gonzalez-Burgos et al., 2019; Amunts et al., 2020).

Thus, the observed improvement in phonemic fluency may indicate that multidimensional, movement-based training can positively influence language processes dependent on executive and speed-related mechanisms (Khalili et al., 2024). Although prior studies describe semantic fluency as stable, our findings partly challenge this view. However, semantic fluency did not show consistent intervention effects at the group level, and the observed within-group improvement among women should be interpreted as exploratory. Male participants in the control group showed declines in semantic fluency, whereas intervention group performance remained stable. Although not statistically significant, this trend could suggest that regular activity helps counteract declines in semantic–associative processes, especially in men.

Physical activity has been associated with greater functional connectivity in fronto-temporal and cerebellar networks relevant for language. Interventions have also demonstrated increases in phonemic fluency and prefrontal–temporal connectivity after walking training (Won et al., 2021). Previous research suggests that men and women may differ in their cognitive responsiveness to exercise interventions, potentially due to differences in baseline activity levels, training engagement, and neurobiological mechanisms (Barha et al., 2017). Our findings may therefore suggest that multidimensional programs could help stabilize semantic fluency in men, although this interpretation requires confirmation in larger samples.

Gender-specific patterns further align with meta-analytic evidence showing that women generally outperform men in phonemic fluency, while no consistent differences exist for semantic fluency (Hirnstein et al., 2023). In our study, women in the intervention group improved in both domains, suggesting that physical activity might strengthen executive–frontal and semantic–associative networks, possibly via enhanced neural connectivity and increased brain-derived neurotrophic factor (BDNF) levels (Erickson et al., 2019; Won et al., 2021). Notably, the within-group improvement in semantic fluency observed among women is particularly interesting in light of the general assumption that semantic processes are relatively stable and less amenable to training. This finding suggests that multidimensional exercise interventions may induce subtle semantic adaptations in women. Overall, these findings imply that regular activity may influence language functions in older age through neurobiological mechanisms such as plasticity, neurotrophic signaling, and network stabilization—manifesting differently across genders.

### Gender-specific effects and underlying mechanisms

The gender-specific differences observed here underscore the need for personalized prevention strategies. Previous research has shown that men and women follow distinct cognitive aging trajectories and respond differently to interventions (Levine et al., 2021; Venneri et al., 2024; Krumpolt et al., 2025). Our findings suggest that interventions addressing complex executive processes might particularly suit men, whereas programs emphasizing divided attention and accuracy could be more effective for women.

The observed improvement in consciously controlled attention might reflect enhanced efficiency within fronto-parietal networks and improved functional connectivity. Multidimensional training could foster neuroplasticity, perfusion, and synaptic adaptation (Erickson et al., 2019), thereby supporting adaptive neural changes. Furthermore, the group-based recreational setting may have enhanced sustained engagement and motivation, thereby supporting adherence and cognitive involvement beyond the physiological effects of exercise alone. The combination of coordinative complexity, cognitive demands, and social interaction may thus constitute an intervention-specific pathway contributing to improved fronto-parietal efficiency and reduced cognitive–motor interference. While these interpretations remain speculative in the absence of neurophysiological measures in the present study, they are consistent with emerging evidence suggesting that multidimensional interventions engaging both motor and cognitive domains may promote adaptive changes in functional brain networks in older adults. For example, recent multidomain intervention research has reported increased functional connectivity within fronto-parietal executive networks following lifestyle interventions in older populations, supporting the assumption of neuroplastic adaptations at the network level (Rolandi et al., 2025). In addition, multidimensional exercise concepts integrating physical, cognitive, and psychosocial components have been proposed as particularly promising approaches for supporting cognitive health in ageing populations (Chang et al., 2022). Together, these findings with fMRI provide a plausible neurobiological framework for interpreting the present results, even though direct neuroimaging evidence was not obtained in this study.

## Conclusion

A six-month multidimensional exercise program improved executive and divided attention as well as phonemic verbal fluency in previously inactive older adults aged 65–70, whereas semantic fluency and simple reaction time remained largely stable. Gender-specific effects were observed: men showed greater gains in consciously controlled attention and phonemic fluency, while women enhanced accuracy and reduced dual-task costs. These findings underline that cognitive effects of physical activity are both domain- and gender-dependent. Incorporating executive and dual-task elements into regular community-based programs could effectively strengthen everyday functioning and cognitive resilience in aging. Such multidomain interventions represent a feasible and motivating strategy for older adults with different prior activity levels. Future research should explore the neural mechanisms underlying these changes and evaluate the long-term sustainability of the observed benefits to guide the development of equitable, evidence-based exercise programs for healthy cognitive aging.

### Limitations

Several limitations should be considered when interpreting the present findings. First, although a controlled design was applied, baseline differences between the intervention and control groups were observed for some cognitive parameters. Consequently, significant interaction effects—particularly higher-order interactions involving time, group, and sex—should be interpreted with caution, as pre-existing group differences may have influenced the magnitude and direction of observed changes. Second, the control group was relatively small compared with the intervention group, and the overall sample was characterized by an unequal sex distribution, with a higher proportion of women than men. While linear mixed models were used to account for unequal group sizes and repeated measurements, limited subgroup sizes—especially among men in the control group—may have reduced statistical power and constrained the robustness of gender-specific conclusions. Third, the generalizability of the findings is limited by the regional recruitment strategy. Participants were recruited from a single metropolitan area and were willing to engage in a structured exercise program, which may restrict the transferability of results to other regions, rural settings, or populations with different socio-cultural or health-related characteristics. Fourth, although the control group was instructed to maintain their usual lifestyle, treatment contamination cannot be fully excluded. Participants in the control group may have increased their physical activity or cognitive engagement during the study period due to increased awareness or informal participation in activities outside the study context, potentially attenuating between-group differences. Fifth, this study used a non-randomized controlled design for logistical reasons, which limits causal inference and means that baseline differences and residual confounding cannot be fully ruled out. Finally, the study focused on short-term pre–post effects following a six-month intervention. Long-term sustainability of the observed cognitive changes could not be assessed, and future studies should include follow-up measurements to determine whether the benefits persist beyond the intervention period. The last point, the relatively high educational background of the sample may also reflect increased cognitive reserve and health awareness, which are known to be associated with more favorable health behaviors and a greater willingness to engage in structured interventions. Although no participants were excluded based on educational level and all individuals meeting the inclusion criteria were eligible to participate, it is likely that the study did not equally reach individuals with lower educational attainment (Liu et al., 2023). This potential self-selection bias should be considered when interpreting the generalizability of the findings.

## Data Availability

The raw data supporting the conclusions of this article will be made available by the authors, without undue reservation.
